# Biofilm Eradication by Symmetrical Selenoesters for Food-Borne Pathogens

**DOI:** 10.3390/microorganisms8040566

**Published:** 2020-04-15

**Authors:** Márta Nové, Annamária Kincses, Beatrix Szalontai, Bálint Rácz, Jessica M. A. Blair, Ana González-Prádena, Miguel Benito-Lama, Enrique Domínguez-Álvarez, Gabriella Spengler

**Affiliations:** 1 Department of Medical Microbiology and Immunobiology, Faculty of Medicine, University of Szeged, Dóm tér 10, 6720 Szeged, Hungary; nove.marta@med.u-szeged.hu (M.N.); kincses.annamaria@med.u-szeged.hu (A.K.); beatrixszalontai@gmail.com (B.S.); balintracz95@gmail.com (B.R.); 2 Institute of Microbiology and Infection, College of Medical and Dental Sciences, University of Birmingham, Birmingham B15 2TT, UK; J.M.A.Blair@bham.ac.uk; 3 Instituto de Química Orgánica General (IQOG-CSIC), Consejo Superior de Investigaciones Científicas, Juan de la Cierva 3, 28006 Madrid, Spain; anagonzalez.prad@gmail.com (A.G.-P.); m.benito@iqog.csic.es (M.B.-L.)

**Keywords:** *Salmonella* species, *Staphylococcus aureus*, multidrug resistance, antibacterial activity, symmetrical selenoesters

## Abstract

Infections caused by *Salmonella* species and *Staphylococcus aureus* represent major health and food industry problems. Bacteria have developed many strategies to resist the antibacterial activity of antibiotics, leading to multidrug resistance (MDR). The over-expression of drug efflux pumps and the formation of biofilms based on quorum sensing (QS) can contribute the emergence of MDR. For this reason, the development of novel effective compounds to overcome resistance is urgently needed. This study focused on the antibacterial activity of nine symmetrical selenoesters (Se-esters) containing additional functional groups including oxygen esters, ketones, and nitriles against Gram-positive and Gram-negative bacteria. Firstly, the minimum inhibitory concentrations of the compounds were determined. Secondly, the interaction of compounds with reference antibiotics was examined. The efflux pump (EP) inhibitory properties of the compounds were assessed using real-time fluorimetry. Finally, the anti-biofilm and quorum sensing inhibiting effects of selenocompounds were determined. The methylketone and methyloxycarbonyl selenoesters were the more effective antibacterials compared to cyano selenoesters. The methyloxycarbonyl selenoesters (**Se-E2** and **Se-E3**) showed significant biofilm and efflux pump inhibition, and a methyloxycarbonyl selenoester (**Se-E1**) exerted strong QS inhibiting effect. Based on results selenoesters could be promising compounds to overcome bacterial MDR.

## 1. Introduction

The emergence of multidrug resistant pathogens is a major problem, leading to a progressive reduction in the efficiency of many antibacterial agents. This phenomenon is a serious challenge in public healthcare and medicine [1].

The most frequent multidrug resistance (MDR) mechanisms enable the resistant bacteria to achieve one or several of the following effects: (a) limited uptake of drug; (b) target modification; (c) drug inactivation; and (d) active efflux mediated by efflux pumps. Some efflux pumps are expressed constitutively, whereas others are induced or over-expressed under environmental stimuli [2]. There are six families of the efflux pump systems: ATP-binding cassette (ABC) family, multidrug and toxic compound extrusion (MATE) family, small multidrug resistance (SMR) family, major facilitator family (MFS), resistance nodulation division (RND) family, and proteobacterial antimicrobial compound efflux (PACE) family [3,4]. Gram-positive bacteria mainly express the members of the MATE and MFS families, whereas Gram-negative bacteria also have transporters of the RND family [2]. The AcrAB-TolC efflux system is comprised of AcrB which belongs to the RND efflux transporters, the outer membrane protein TolC, and the periplasmic adaptor protein AcrA [5].

The formation of biofilms can also contribute to bacterial resistance. Biofilms have a dynamic structure involving a multicellular bacterial community and an extracellular polymeric matrix produced by the bacterial population. Biofilm-associated infections can lead to antibiotic resistant and persistent infections as this environment enhances the ability of the embedded bacteria to resist the action of the antibiotics [6].

One of the major food-borne illnesses is the salmonellosis caused by non-typhoidal *Salmonella enterica* [7]. In addition, the staphylococcal food poisoning (SFP) is a frequent food-born disease caused by staphylococcal enterotoxin (SE) producer enterotoxigenic *Staphylococcus aureus* strains [8]. *S. aureus* and *Salmonella enterica* serovar Typhimurium are food-borne pathogens capable of forming biofilms on various surfaces. Alkaline and acidic detergents, as well as iodophores, can be effective against biofilm. However, these substances damage surfaces, and the inappropriate use of biocides and disinfectants could lead to a quick and undesired emergence of resistant microbes [9]. Many bacteria use a cell–cell communication system, namely quorum sensing (QS), to coordinate the population density-dependent gene expression pattern [10]. This communication system plays a major role in biofilm development, as bacteria can produce new virulence factors and thanks to them this bacterial community responds poorly to antibiotic treatment [11].

Selenium(Se)-containing compounds could provide alternative and effective scaffolds to overcome MDR [12]. Se is an essential trace element in living organisms and is crucial for the nutrient supply and energy generation of bacteria. However, overdoses of Se can be highly toxic [13,14]. There is significant evidence about the pro-oxidant effect of Se, particularly in the form of sodium selenite (Na_2_SeO_3_), while selenomethionine and selenocysteine are less toxic [14]. It has been described previously that Se-containing agents have an antibacterial effect [15,16]. Selenoesters and selenoanhydrides have exhibited anti-biofilm activity against *S. aureus* and *S*. Typhimurium as described previously [17]. Furthermore, selenocompounds have been used as selenium nanoparticles (SeNPs) against *S. aureus*, *Escherichia coli*, and *Pseudomonas aeruginosa* strains [18,19].

In the present study, and based in these antecedents, symmetrical 2-oxopropyl selenoesters, methyloxycarbonylmethyl selenoesters, and methylcyano selenoesters have been investigated against Gram-negative and Gram-positive bacterial strains to determine their antibacterial, efflux pump inhibiting, and anti-biofilm properties.

## 2. Materials and Methods

### 2.1. Compounds

**Scheme 1:**
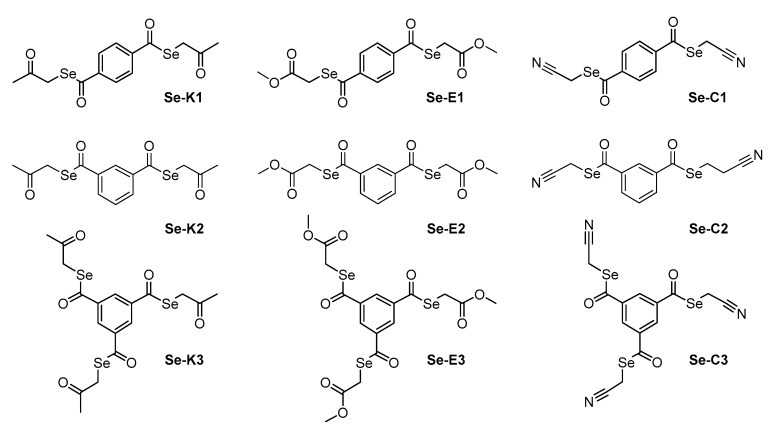
Chemical structure of the symmetrical selenoesters evaluated.

Nine symmetrical selenodiesters or selenotriesters were synthesized and evaluated. Three were 2-oxopropyl selenoesters (briefly, ketone selenoesters, or methylketone selenoesters; compounds **Se-K1**, **Se-K2** and **Se-K3**). The next three selenocompounds were methyloxycarbonylmethyl selenoesters (methylcarbonyl selenoesters or methyloxycarbonyl selenoesters; compounds **Se-E1**, **Se-E2,** and **Se-E3**) [20]. The final three compounds were methylcyano selenoesters (cyano selenoesters; compounds **Se-C1**, **Se-C2,** and **Se-C3**). For each group of three compounds, the first is the symmetrical para-disubstituted derivative, the second is the symmetrical meta-substituted derivative, and the third is the symmetrical 1,3,5-trisubstituted derivative (Scheme 1). Their synthesis is described in the patent application EP17382693, and they were adequately characterized using nuclear magnetic resonance spectroscopy (NMR), mass spectrometry (MS), and infrared spectroscopy (IR) techniques and their purity was assessed by elemental analysis [21]. Before their use in biological assays the selenocompounds were dissolved in dimethyl sulfoxide (DMSO), to obtain 10 mM concentration stock solutions.

### 2.2. Reagents and Media

DMSO (Sigma-Aldrich, St Louis, MO, USA), phosphate-buffered saline (PBS; pH 7.4), promethazine (PMZ; EGIS), verapamil, carbonyl cyanide *m*-chlorophenyl hydrazone (CCCP), ethidium bromide (EB), ciprofloxacin-hydrochloride (CIP) tetracycline-hydrochloride (TET), crystal violet (CV), Luria-Bertani (LB) broth, and LB agar were purchased from Sigma-Aldrich Chemie GmbH (Steinheim, Germany). The modified LB agar (LB*) was prepared from bacteriological agar 20 g/L (Difco, Detroit, USA), tryptone 10 g/L, NaCl 10 g/L, yeast extract 5 g/L, K_2_HPO_4_ 1 g/L, MgSO_4_ × 7H_2_O 0.3 g/L, and FeNaEDTA 36 mg/L. pH of the agar was adjusted to 7.2. Mueller–Hinton (MH) broth, tryptic soy broth (TSB), and tryptic soy agar was purchased from Scharlau Chemie S.A. (Barcelona, Spain).

### 2.3. Bacterial Strains

Compounds were evaluated against the following bacterial strains:

Gram-negative wild-type *Salmonella enterica* serovar Typhimurium SL1344 (SE01) expressing the AcrAB-TolC pump system and its *acrB* gene inactivated mutant *S*. Typhimurium SL1344 strain (SE02), *acrA* gene inactivated mutant *S*. Typhimurium SL1344 (SE03), and *tolC* gene inactivated mutant *S*. Typhimurium SL1344 strain (SE39) were used in the study [22,23,24,25].

Gram-positive *Staphylococcus aureus* American Type Culture Collection (ATCC) 25923 was used as the methicillin-susceptible reference bacterial strain, and the methicillin and ofloxacin-resistant *S. aureus* 272123 clinical isolate (MRSA), which was kindly provided by Prof. Dr. Leonard Amaral (Institute of Hygiene and Tropical Medicine, Lisbon, Portugal), was used in the assays.

For QS tests we used *Chromobacterium violaceum* 026 (CV026) as a sensor strain and *Enterobacter cloacae* 31298 as a N-acyl-homoserine lactone (AHL) producer clinical bacterial isolate. If *C. violaceum* reaches a high cell density, it produces violacein, which is a purple pigment [26,27].

### 2.4. Cell Line

MRC-5 human embryonal lung fibroblast cell line (ATCC CCL-171) was purchased from LGC Promochem, Teddington, UK. The cells were cultured in Eagle’s Minimal Essential Medium (EMEM, containing 4.5 g/L glucose) supplemented with a non-essential amino acid mixture, a selection of vitamins, and 10% heat-inactivated fetal bovine serum. The cell lines were incubated at 37 °C, in a 5% CO_2_, 95% air atmosphere.

### 2.5. Determination of Minimum Inhibitory Concentrations by Microdilution Method

The minimum inhibitory concentrations (MICs) of compounds were determined according to the Clinical and Laboratory Standard Institute guidelines (CLSI) [28]. MIC values of the compounds were determined by visual inspection. The solvent was also assayed to ensure there was no antibacterial effect and the concentration (1 *v/v*%) applied in the assays had no antibacterial activity. DMSO was used at subinhibitory concentration (1 *v/v*%) in the assays.

### 2.6. Cytotoxicity Assay

The adherent MRC-5 human embryonal lung fibroblast cells were cultured in 96-well flat-bottomed microtiter plates, using EMEM supplemented with 10% heat-inactivated fetal bovine serum. The density of the cells was adjusted to 1 × 10^4^ cells in 100 μL per well, the cells were seeded overnight at 37 °C, 5% CO_2_, then the medium was removed from the plates containing the cells, and the dilutions of selenocompounds previously made in a separate plate were added to the cells in 200 μL.

The culture plates were incubated at 37 °C for 24 h; at the end of the incubation period, 20 μL of MTT (thiazolyl blue tetrazolium bromide, Sigma) solution (from a stock solution of 5 mg/mL) was added to each well. After incubation at 37 °C for 4 h, 100 μL of sodium dodecyl sulfate (SDS; Sigma) solution (10% in 0.01 M HCI) was added to each well and the plates were further incubated at 37 °C overnight. Cell growth was determined by measuring the optical density (OD) at 540/630 nm with Multiscan EX ELISA reader (Thermo Labsystems, Cheshire, WA, USA). Inhibition of the cell growth was determined according to the formula below:

IC_50_ = 100 − [(OD_sample_ − OD_medium control_)/(OD_cell control_ − OD_medium control_)] × 100 (1)

Results are expressed in terms of IC_50_, defined as the inhibitory dose that reduces the growth of the cells exposed to the tested compounds by 50%.

### 2.7. Resistance Modulation Assay

The resistance modulation effect of compounds with ciprofloxacin (CIP) and tetracycline (TET) antibiotics were evaluated by the checkerboard method on *S. aureus* strains. Briefly, CIP or TET was diluted in a 96-well microtiter plate by two-fold serial dilution in MH broth and then the compounds were added at subinhibitory concentrations (½ MIC). In this assay, only the tested compounds with well-defined MIC values were tested. Finally, 10^−4^ dilution of the overnight bacterial culture in MH was added to each well. The final volume was 200 µL in each well. The microtiter plates were incubated at 37 °C for 18 h. MIC values in the presence of the antibiotics alone and in combination with Se-compounds were determined by visual inspection.

### 2.8. Real-Time Ethidium Bromide Accumulation Assay

The impact of compounds on EB accumulation was determined by the automated EB method using a CLARIOstar Plus plate reader (BMG Labtech, UK). Firstly, the bacterial strain was incubated until it reached an optical density (OD) of 0.6 at 600 nm. The culture was washed with phosphate buffered saline (PBS; pH 7.4) and centrifuged at 13,000× *g* for 3 min, the cell pellet was re-suspended in PBS. The compounds were added at ½ MIC concentration to PBS containing a non-toxic concentration of EB (1 µg/mL). Then, 50 μL of the EB solution containing the compound were transferred into 96-well black microtiter plate (Greiner Bio-One Hungary Kft, Hungary), and 50 μL of bacterial suspension (OD_600_ 0.6) were added to the each well. Then, the plates were placed into the CLARIOstar plate reader, and the fluorescence was monitored at excitation and emission wavelengths of 530 nm and 600 nm every minute for one hour on a real-time basis. From the real-time data, the activity of the compounds, namely the relative fluorescence index (RFI) of the last time point (minute 60) of the EB accumulation assay, was calculated according to the following formula:

RFI = (RF_treated_ − RF_untreated_)/RF_untreated_ (2)

where RF_treated_ is the relative fluorescence (RF) at the last time point of EB retention curve in the presence of an inhibitor, and RF_untreated_ is the RF at the last time point of the EB retention curve of the untreated control having the solvent control (DMSO).

### 2.9. Measuring Biofilm Formation Using Crystal Violet

The anti-biofilm effect of the tested compounds against *S. aureus* strains and wild-type *S.* Typhimurium SE01 was measured using crystal violet (CV; 0.1% (*v/v*)). This dye is used to detect the total biofilm biomass formed. Overnight cultures were diluted to OD of 0.1 at 600 nm in TSB medium. Then, the bacterial cultures were added to 96-well microtiter plates and the compounds were added at ½ MIC concentration. The final volume was 200 μL in each well. The microtiter plates were incubated at 30 °C for 48 h with gentle agitation (100 rpm). After the incubation period, TSB medium was discarded, and the plates were washed with tap water to remove unattached cells. Then 200 μL crystal violet was added to the wells and incubated for 15 min at room temperature. Then, CV was removed from the wells and the plates were washed again with tap water, and 200 μL of 70% ethanol was added to the wells. Finally, the biofilm formation was determined by measuring the OD at 600 nm using Multiscan EX ELISA plate reader (Thermo Labsystems, Cheshire, WA, USA). The anti-biofilm effect of compounds was expressed in the percentage (%) of decrease in biofilm formation.

### 2.10. Quorum Sensing (QS) Assay

The QS inhibitory effect of selenocompounds was examined on the AHL producer *E. cloacae* strain and *C. violaceum* sensor bacterial strain. These strains were inoculated in parallel. The QS inhibition was monitored by agar diffusion method on LB* agar plate as described previously [29]. Filter paper discs (7.0 mm in diameter) were placed between the parallel inoculated strains and impregnated with 10 μL compounds. Starting concentration of the compounds was ½ MIC. The agar plates were incubated at room temperature (20 °C) for 24–48 h and the inhibition of violacein production was measured.

### 2.11. Statistical Analysis

The values are given as the mean ± standard deviation (SD) determined for three replicates from three independent experiments. The analysis of data was performed using SigmaPlot for Windows Version 12.0 software (Systat Software Inc, San Jose, CA, USA), applying the two-tailed *t*-test.

## 3. Results

### 3.1. Determination of Minimum Inhibitory Concentrations by Microdilution Method

Based on the MIC values, the Se-compounds were more effective against *S. aureus* strains. The most effective compounds were the ketone selenoesters **Se-K1**, **Se-K2**, and **Se-K3** on the reference *S. aureus* ATCC 25923, showing an MIC of 0.39 μM. Interestingly, these three derivatives share a common moiety, namely a methylketone group in the alkyl moiety bound to the selenium atom. The replacement of this methylketone by a cyano or by a methyloxycarbonyl moiety reduced the activity dramatically, as the MICs were 16- and 32-fold higher against *S. aureus* ATCC 25923, respectively; with the exception of the trisubstituted derivative **Se-C3**, as its MIC was only 4-fold higher than the MIC of the trisubstituted methylketone **Se-K3**. The same tendency, but accentuated, was observed in *S. aureus* MRSA 272123, where the MIC values of the methylketone derivatives were in the range of 64- to 128-fold lower than the equivalent methyloxycarbonyl derivatives and in the range of 16- to 32-fold lower than the equivalent nitrile-containing selenoesters. The compounds showed a slight antibacterial effect on *Salmonella* strains. The most effective compound was **Se-C3** on SE01, SE02, and SE03 strains, showing an MIC of 12.5 µM (Table 1). Importantly, the MIC to the efflux knockout strains was unchanged suggesting that the compounds were not substrates of the AcrAB-TolC efflux pump.

### 3.2. Resistance Modulation Assay

As the Se-compounds were more effective on *S. aureus* strains, these strains were selected for combination studies with reference antibiotics. Selenocompound **Se-E3** showed synergism with TET on the methicillin-susceptible *S. aureus* ATCC 25923.

Surprisingly, all selenocompounds showed synergism with TET on the methicillin-resistant *S. aureus* strain. **Se-E3** and **Se-C2** were the most effective compounds in combination with TET, as they reduced the MIC value of TET against this MRSA strain to a value 32-fold lower. Additionally, compounds **Se-E1** and **Se-C1** also exerted a noteworthy reduction of the MIC value, of 16-fold in this case. On the other hand, **Se-K1** and **Se-E3** showed synergism with CIP on the MRSA strain, achieving a 2-fold reduction of the MIC value (Table 2).

### 3.3. Ethidium Bromide Accumulation Assay

The activity of the selenocompounds on EB accumulation was determined by the automated EB method on sensitive and resistant *S. aureus* and *S.* Typhimurium SE01, -02, -03, and -39 strains. The relative fluorescence index was calculated based on the means of relative fluorescence units (RFUs; Table 3).

**Figure 1:**
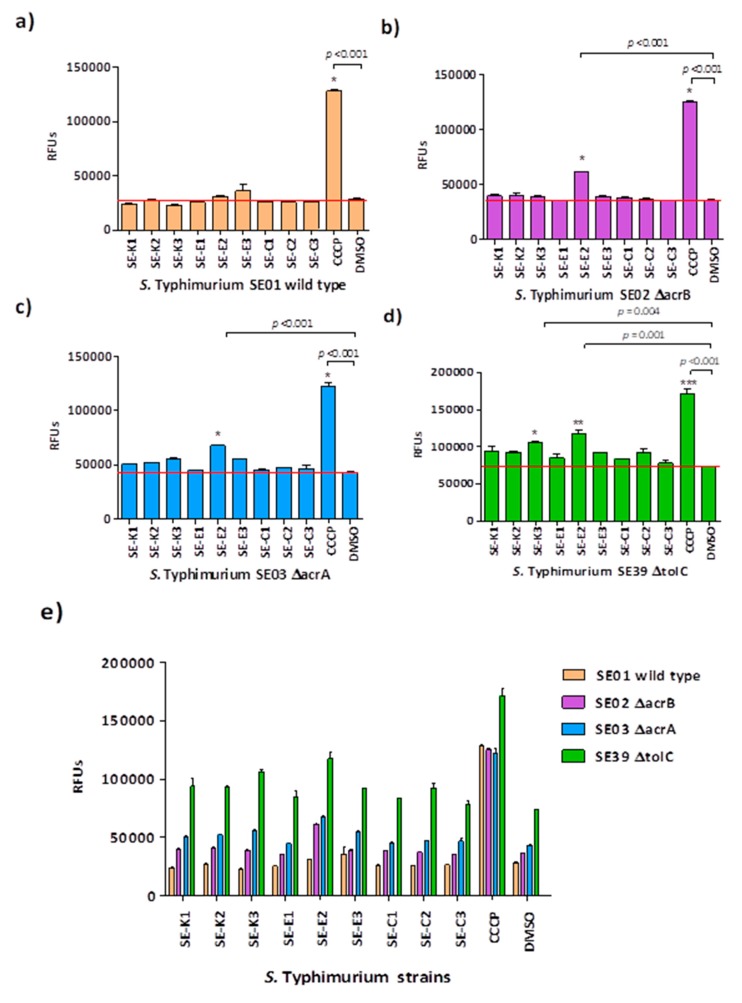
Ethidium bromide (EB) accumulation in *S*. Typhimurium strains in the presence of Se-compounds. The graphs show the relative fluorescence units (RFUs) of (**a**) *S.* Typhimurium SE01, (**b**) *S.* Typhimurium SE02, (**c**) *S.* Typhimurium SE03, (**d**) *S.* Typhimurium SE39, and (**e**) all *S.* Typhimurium bacterial strains in the presence of the compounds in the 60th minute of the assay. In case of *S.* Typhimurium SE01, -SE02 and -SE03 the level of significance was * *p* < 0.001. The levels of significance were * *p* = 0.004, ** *p* = 0.001, and *** *p* < 0.001 on *S.* Typhimurium SE39.

In case of *Salmonella* strains, the Se-compounds increased the intracellular EB accumulation more efficiently on the *tolC* gene inactivated mutant *S*. Typhimurium SE39 after 60 min. In contrast, RFUs obtained in the presence of Se-compounds were the lowest on the wild-type *S*. Typhimurium SE01. CCCP, the reference efflux pump inhibitor (EPI) was the positive control in case of *Salmonella* and reference *S. aureus* strain. In addition, verapamil was applied as reference EPI on *S. aureus* MRSA. The solvent DMSO served as a negative control in the experiments. **Se-E2** significantly increased the intracellular EB accumulation on *S*. Typhimurium SE02, -03, and -39. In addition, a significant EB accumulation was observed for **Se-K3** on *S*. Typhimurium SE39 (Figure 1).

**Figure 2:**
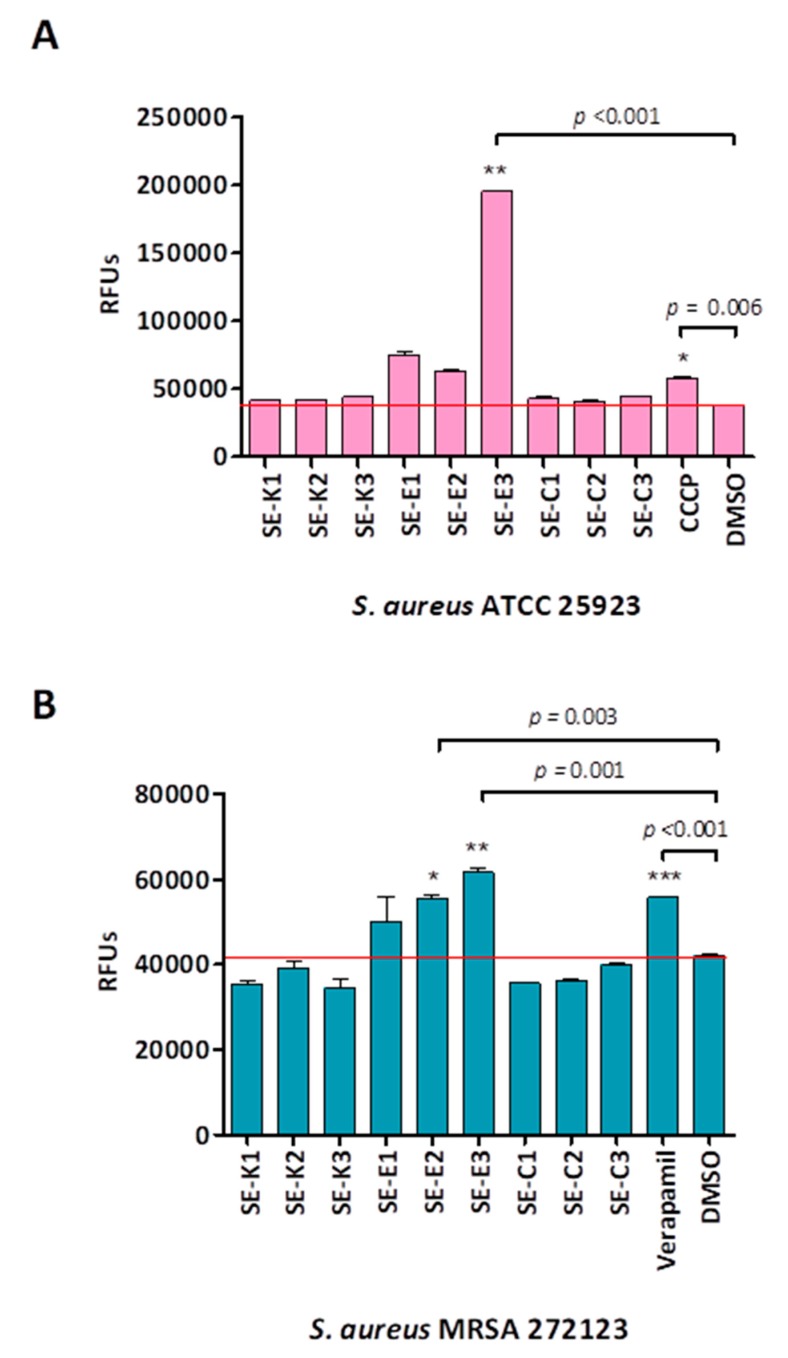
EB accumulation on *S. aureus* strains. The graphs show the RFUs of (**A**) *S. aureus* ATCC 25923 (**B**) *S. aureus* MRSA 272123 bacterial strains in the presence of the compounds in the 60th minute of the assay. In case of *S. aureus* ATCC 25923 the levels of significance were * *p* = 0.006 and ** *p* < 0.001. The levels of significance were * *p =* 0.003, ** *p* = 0.001, and *** *p* < 0.001 on *S. aureus* MRSA 272123.

In case of the reference *S. aureus* and resistant MRSA strain the highest RFUs were recorded in the presence of **Se-E3**, for this reason this compound exerted the most prominent EPI activity. In addition, methylcarbonyl selenoesters **Se-E1** and **Se-E2** were proven to be effective in both *S. aureus* strains (Figure 2).

### 3.4. Measuring Biofilm Formation Using Crystal Violet

**Figure 3:**
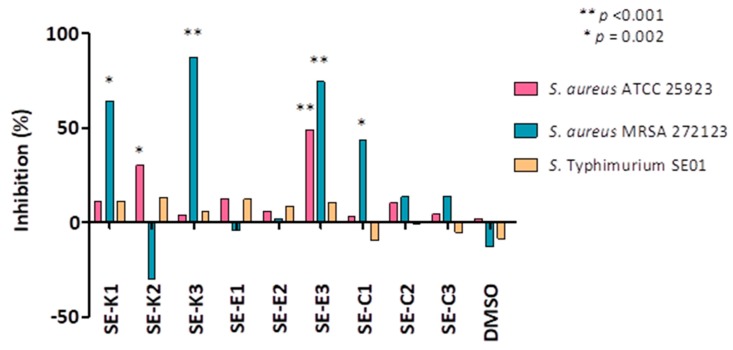
Anti-biofilm effect of Se-compounds on *S*. Typhimurium SE01 wild-type and on sensitive and resistant *S. aureus* strains. The levels of significance were ** *p* < 0.001 and * *p* = 0.002, respectively.

The effect of selenocompounds on biofilm formation of sensitive and resistant *S. aureus* strains and wild-type *S.* Typhimurium SE01 was evaluated. The biofilm inhibition (%) was calculated based on the mean of absorbance units (AUs). The absorbance expressed in AUs was the following on non-treated samples: reference *S. aureus* showed an absorbance of 2.4 ± 0.1, the resistant *S. aureus* exhibited 1.3 ± 0.1 AU, and the wild-type *S.* Typhimurium presented 2.2 ± 0.3 AU. Selenocompounds **Se-K1** (AU: 0.45 ± 0.17; inhibition: 64.5%), **Se-K3** (AU: 0.16 ± 0.06; inhibition: 84.7%), **Se-E3** (AU: 0.32 ± 0.07; inhibition: 74.6%), and **Se-C1** (AU: 0.72 ± 0.15; inhibition: 43.7%) could efficiently inhibit the biofilm formation of *S. aureus* MRSA. In case of the reference *S. aureus* strain, the anti-biofilm effect was observed for **Se-K2** (AU: 1.67 ± 0.10; inhibition: 30.3%) and **Se-E3** (AU: 1.22 ± 0.17; inhibition: 74.6%). The compounds showed no significant anti-biofilm effect on *S.* Typhimurium SE01 (Figure 3).

### 3.5. Quorum Sensing (QS) Assay

**Figure 4:**
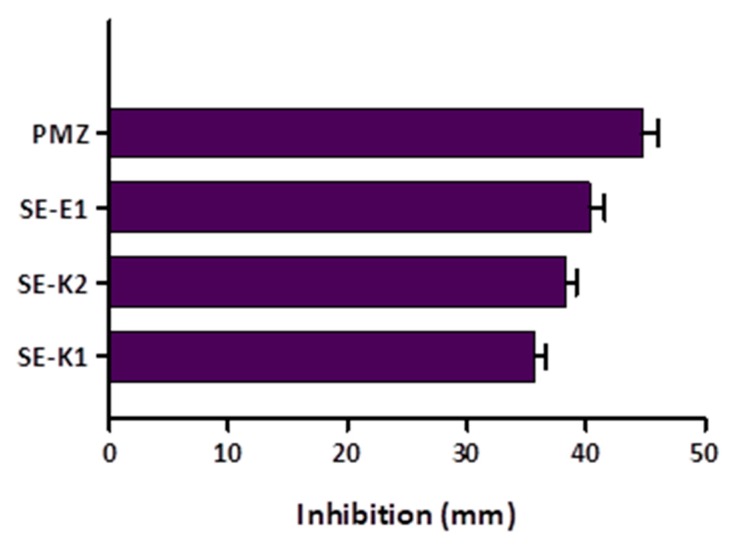
Quorum Sensing (QS) inhibition by selenocompounds. The QS-inhibition assay was performed using the parallel inoculation disk diffusion method. The ineffective compounds are not shown. Promethazine (PMZ) was used as a positive control.

The sensor strain *C. violaceum* 026 and the AHL producer strains *E. cloacae* 31298 were inoculated as parallel lines. Interactions between the strains and compounds were evaluated for the reduction in the size of the zone of pigment production and the zone of growth inhibition of the affected strains, in millimeters. Promethazine (PMZ) was applied as a QS inhibitor and its zone of inhibition was 46 mm. Selenocompounds **Se-K1**, **Se-K2**, and **Se-E1** had QS inhibitory effect. In addition, **Se-K1** and **Se-K2** showed inhibition zones of 37 mm and 40 mm, respectively, whereas the methyloxycarbonyl selenoester **Se-E1** was the most effective QS inhibitor with an inhibition zone of 41 mm (Figure 4).

### 3.6. Cytotoxicity Assay on Normal Human Fibroblasts

In order to determine the toxicity and safety of the selenocompounds on human cells, a cytotoxicity assay was performed using normal MRC-5 human embryonal lung fibroblast cells (Table 4).

Based on the data obtained, ketone selenoesters **Se-K1**, **Se-K2**, and **Se-K3** presented high toxicity on normal cells (IC_50_ between 0.5 and 1.5 µM). Fortunately, the methylcarbonyl selenoesters (**Se-E1**, **Se-E2**, and **Se-E3**) and the cyano selenoesters (**Se-C1**, **Se-C2**, and **Se-C3**) showed no toxicity on normal cells as all their IC_50_ values were above 75 µM.

## 4. Discussion

In case of MIC determination, the symmetrical selenoesters evaluated herein (whose selenium-bound alkyl moiety contains functional groups as a ketone, oxygen ester or nitrile) were more effective on sensitive and resistant *S. aureus* strains compared to the four *S.* Typhimurium bacterial strains. This suggests that these symmetrical selenoesters are more active against Gram-positive bacteria (as *Staphylococcus aureus*) than against Gram-negative bacteria (as *Salmonella enterica* serovar Typhimurium). This fact is in accordance with the antibacterial activity of non-symmetrical selenoesters, which were evaluated in a previous work of the group [27]; only three non-symmetrical ketone selenoesters (**9–11** in [27]) were active against *S. aureus*, whereas none of them were active against *Escherichia coli*. Interestingly, all of them were active against *Chlamydia trachomatis* (Gram-negative), but since *Chlamydia* is an intracellular bacterium this may affect its sensitivity to the compounds [27].

The methylketone selenoesters **Se-K1**, **Se-K2**, and **Se-K3** were the most potent antibacterials on reference *S. aureus*. In contrast, the methyloxycarbonyl selenoesters **Se-E1**, **Se-E2**, and **Se-E3** and the cyano selenoesters **Se-C1** and **Se-C2** showed strong resistance modulating activity with tetracycline against the MRSA strain. Comparing the antibacterial activity with the previously reported data [27], two observations are of interests. First, the symmetrical selenoesters are more potent antibacterials against *S. aureus* ATCC 25923 than the respective asymmetrical derivatives. This is observed when we compare the 0.39 μM MIC values of **Se-K1**, **Se-K2**, and **Se-K3** with the 3.12 μM MIC value of **9** in [27] (methylketone selenoesters), and the 12.5 μM MIC values of **Se-E1**, **Se-E2** and **Se-E3** with **7** in [27], which was not active at concentrations below 100 μM (methyloxycarbonyl selenoesters). Second, the symmetrical methyl selenoesters **2–5** in [27] were not active against *S. aureus* ATCC 25923 (MIC > 100 μM), whereas all the functionalized selenoesters evaluated in this work (-CH_2_COCH_3_, -CH_2_COOCH_3_, -CH_2_CN) showed MIC values against this strain at 12.5 μM or lower. This indicates that these second-generation selenoesters have improved antibacterial activity compared with those that have been previously reported.

If we compare the antibacterial activity of the symmetrical selenocompounds with its toxicity against MRC-5 normal embryonal lung fibroblast cell line, we observe that the MIC values of the compounds against *S. aureus* ATCC 25923 were lower than the IC_50_ values against this cell line.

In the resistance modulation assay, the selenocompounds were tested at ½ of their MIC in combination with tetracycline and ciprofloxacin in the two *S. aureus* strains (ATCC 25923 and MRSA 272123). As mentioned previously, all compounds were able to modulate the activity of tetracycline against *S. aureus* MRSA 272123. The results were somehow comparable with the antibacterial activity. Interestingly, the –CH_2_COOCH_3_ and –CN containing symmetrical selenoesters were more potent modulators than the –CH_2_COCH_3_ selenoesters (X-fold reductions of 2–4, 8–32, and 4–32, respectively). However, as MIC values of the selenocompounds were higher against this *S. aureus* strain, only **Se-C1** and **Se-C2** could be used at a safe concentration (25 μM, non-toxic in MRC-5 cells) with a noteworthy effect (16- and 32-fold reduction of MIC value of tetracycline).

Real-time EB accumulation was applied in order to monitor the EPI activity of the compounds. The intracellular EB accumulation was the highest on the *tolC* gene inactivated mutant *S*. Typhimurium SE39, and the lowest EB accumulation was obtained in the wild-type *S*. Typhimurium SE01 in the presence of methyloxycarbonyl selenoester **Se-E2**. This compound significantly increased the EB accumulation in the efflux pump gene inactivated (*ΔacrA*, *ΔacrB*, and *ΔtolC*) mutant *S*. Typhimurium strains due to efflux independent mechanisms, e.g., membrane destabilizing effect. In addition, methyloxycarbonyl selenoester **Se-E3** showed significantly effective pump inhibition on sensitive (*p* < 0.001) and resistant (*p* = 0.001) *S. aureus* strains. Unfortunately, these two Se-compounds have to be applied at a high concentration (50 μM, which is ½ of their MIC) against *S.* Typhimurium (**Se-E2**) or *S. aureus* MRSA 272123 (**Se-E3**), respectively. Compound **Se-E3** could be used in this application against *S. aureus* ATCC 25923, as in this case its concentration would be 6.25 μM, much lower.

Regarding the anti-biofilm effect, the methyloxycarbonyl selenoester **Se-E3** showed significant biofilm inhibition on both of sensitive and resistant *S. aureus* strains. Furthermore, the methylketone selenoester **Se-K3** was the most effective anti-biofilm agent on resistant *S. aureus* MRSA. In addition, **Se-K1** was also interesting, as it showed a biofilm inhibiting effect higher than 50% against MRSA. It was surprising that **Se-K2** promoted the biofilm formation of *S. aureus* MRSA, because it has the same chemical formula as **Se-K1** (both are 2-oxopropyl selenodiesters); they only differ in the substitution pattern at the phenyl ring, such that **Se-K1** has a *para* substitution (1,4) and **Se-K2** has a *meta* substitution (1,3). It is interesting to see how such a small change in the substitution pattern at the core phenyl ring leads to completely different activities. What is more, in **Se-K2** the inclusion of a third –COSeCH_2_COCH_3_ at the position five of the core phenyl ring led to **Se-K3**, recovering the biofilm inhibition in respect to **Se-K2** and enhancing it in respect to **Se-K1**. In the case of the methyloxycarbonyl selenoesters, only the trisubstituted derivative **Se-E3** was capable of significantly inhibiting the biofilm formation in both strains of *S. aureus* (reference and MRSA), whereas the two disubstituted ones were inactive. Methylcyano selenoesters showed a lower inhibition than the other two families of compounds, however, one of them (the *para*-disubstituted (**Se-C1**)) was close to exerting a 50% inhibition of *S. aureus* MRSA.

Finally, QS inhibiting effect of compounds was evaluated based on the inhibition of violacein production. The methylketone selenoester **Se-K1** and **Se-K2** and the methyloxycarbonyl selenoester **Se-E1** were potent QS-inhibitors, with **Se-E1** being the most effective QS inhibitor of these three derivatives by showing an inhibition close to the reference promethazine (positive control).

All these findings reveal that the symmetrical selenoesters have a potent antibacterial activity, mainly against *S. aureus* strains. Furthermore, the methylcyano selenoesters could be used as potential novel antibiotics. Additional studies to evaluate the ADME-Tox properties of these compounds is needed to evaluate their applicability in medicine more in-depth. Besides, the methylketone selenoesters, which are less selective, still could be used, for example, in disinfection of surfaces or in the coating of surfaces to prevent biofilm formation.

## 5. Conclusions

It can be concluded that all the symmetrical selenoesters evaluated have a potent antibacterial activity against *S. aureus* ATCC 25923. The most potent derivatives were the methylketone selenoesters (**Se-K1**, **Se-K2**, and **Se-K3**), followed by the cyano selenoesters (**Se-C1**, **Se-C2**, and **Se-C3**), and at the end by the methyloxycarbonyl selenoesters (**Se-E1**, **Se-E2**, and **Se-E3**). After determining the toxicity on normal fibroblasts, the more selective ones were the cyano selenoesters, followed by the methyloxycarbonyl selenoesters, and the ones by the methylketone selenoesters. Combining both the antibacterial activity and the cytotoxic activity, the most promising compound against *S. aureus* ATCC 25923 was **Se-C3**. The tested selenocompounds also showed antibacterial activity against *S. aureus* MRSA 272123 and against different strains of *S.* Typhimurium, although with higher MIC values.

In addition to the antibacterial activity, the methyloxycarbonyl selenoesters and two cyano selenoesters showed strong resistance reversing activity in the presence of tetracycline against the MRSA strain. Additionally, the methyloxycarbonyl selenoester **Se-E3** was the most effective compound concerning the reversal of resistance, efflux pump inhibition, and anti-biofilm activity on *S. aureus* strains.

## 6. Patents

This work explores the antibacterial activity of compounds covered by the patent EP18382693 [21] (filed on 28 September 2018 by Enrique Domínguez-Álvarez, Gabriella Spengler, Claus Jacob and Carmen Sanmartín) more in-depth.

## Figures and Tables

**Table 1:** Antibacterial activity of selenocompounds. Minimum inhibitory concentrations (MICs) of compounds were determined on reference *Staphylococcus aureus* ATCC (American Type Culture Collection) 25923 and methicillin and ofloxacin-resistant *S. aureus* 272123 (MRSA) strains and *Salmonella* Typhimurium strains.

MIC Determination (µM)						
Compounds	*S. aureus* ATCC 25923	*S. aureus* MRSA 272123	*S.* Typhimurium SE01 Wild-Type	*S.* Typhimurium SE02 *ΔacrB*	*S.* Typhimurium SE03 *ΔacrA*	*S.* Typhimurium SE39 *ΔtolC*
**Se-K1**	0.39	1.56	50	50	50	50
**Se-K2**	0.39	1.56	50	50	50	100
**Se-K3**	0.39	0.78	50	25	25	50
**Se-E1**	12.5	100	>100	>100	>100	>100
**Se-E2**	12.5	100	>100	>100	>100	>100
**Se-E3**	12.5	100	>100	>100	>100	>100
**Se-C1**	6.25	50	25	25	25	25
**Se-C2**	6.25	50	25	25	25	25
**Se-C3**	1.56	12.5	12.5	12.5	12.5	25

**Table 2:** Resistance modulating effect of selenocompounds in the presence of antibiotics on *S. aureus* strains. The resistance modulation effect of Se-compounds with ciprofloxacin (CIP) and tetracycline (TET) antibiotics on the *S. aureus* bacterial strains were evaluated by the checkerboard method.

MIC Reduction (µM) In Brackets, the X-Fold Reduction of MIC Is Presented					
Compounds	*S. aureus* ATCC 25923 *with*			*S. aureus* MRSA 272123 *with*	
	TET	CIP		TET	CIP
−	0.88	1.06		14.06	33.99
**Se-K1**	0.88	1.06		3.51 (4)	16.99 (2)
**Se-K2**	0.88	1.06		7.03 (2)	33.99
**Se-K3**	0.88	1.06		7.03 (2)	33.99
**Se-E1**	0.88	1.06		0.88 (16)	33.99
**Se-E2**	0.88	1.06		1.76 (8)	33.99
**Se-E3**	0.44 (2)	1.06		0.44 (32)	16.99 (2)
**Se-C1**	0.88	1.06		0.88 (16)	33.99
**Se-C2**	0.88	1.06		0.44 (32)	33.99
**Se-C3**	0.88	1.06		3.51 (4)	33.99

**Table 3:** Relative fluorescence indices based on real-time ethidium bromide (EB) accumulation data on *S*. Typhimurium and *S. aureus* strains. The active compounds are presented in bold.

Relative Fluorescence Index (RFI)						
Compounds	*S.* Typhimurium SE01 Wild-Type	*S.* Typhimurium SE02 *ΔacrB*	*S.* Typhimurium SE03 *ΔacrA*	*S.* Typhimurium SE39 *ΔtolC*	*S. aureus* ATCC 25923	*S. aureus* MRSA 272123
**Se-K1**	−0.16	0.10	0.17	0.27	0.1	−0.15
**Se-K2**	−0.04	0.13	0.20	0.26	0.11	−0.07
**Se-K3**	−0.20	0.08	0.28	0.44	0.16	−0.18
**Se-E1**	−0.10	−0.03	0.03	0.15	0.98	0.19
**Se-E2**	0.09	**0.70**	**0.56**	**0.59**	0.67	0.33
**Se-E3**	0.26	0.08	0.27	0.25	**4.15**	**0.47**
**Se-C1**	−0.08	0.06	0.04	0.13	0.14	−0.15
**Se-C2**	−0.10	0.03	0.09	0.25	0.08	−0.13
**Se-C3**	−0.07	−0.02	0.08	0.06	0.18	−0.05
**CCCP**	3.50	2.46	1.81	1.32	0.52	−
**Verapamil**	−	−	−	−	−	0.32

**Table 4:** Cytotoxic activity of selenocompounds on MRC-5 human embryonal fibroblast cells, expressed in Inhibitory Concentration 50 (IC_50_) and with the calculated standard deviation (SD).

Compound	MRC-5	
	IC_50_ (µM)	SD ±
**Se-K1**	0.54	0.00
**Se-K2**	1.34	0.16
**Se-K3**	0.74	0.04
**Se-E1**	77.91	15.86
**Se-E2**	>100	−
**Se-E3**	76.61	9.18
**Se-C1**	>100	−
**Se-C2**	>100	−
**Se-C3**	>100	−
